# Effect of Ventolin on QTc in children with respiratory distress

**DOI:** 10.15171/jcvtr.2016.16

**Published:** 2016-06-28

**Authors:** Mohammad Reza Khalilian, Abbas Fayezi, Mohsen Alisamir, Negar Mohammadi Khorasani

**Affiliations:** ^1^Department of Pediatrics, Shahid Beheshti University of Medical Sciences, Tehran, Iran; ^2^Department of Pediatrics, Ahvaz Jundishapur University of Medical Sciences, Ahvaz, Iran

**Keywords:** Ventolin, Electrocardiogram, Children

## Abstract

***Introduction:*** β2–agonists are first election drugs for the treatment of respiratory disease that may alter cardiac autonomic modulation. The aim of this study was to evaluate the effects of nebulized Ventolin on electrocardiogram, particularly QTc interval to assess the potential arrhythmogenic risks.

***Methods:*** A total of 192 patients between 2 months and 15 years which received nebulized Ventolin were enrolled in this study. Patients were divided into two groups. Electrocardiograms of patients before and after nebulized Ventolin were taken. Differences between two groups were assessed using a paired student’s t test.

***Results:*** There was statistically significant differences in QTc before and after Ventolin in each groups (*P*<0.005).Ventolin effect on QTc interval in both groups did not differ. In first group, there was statistically significant differences between heart rate before and after Ventolin taken (*P*=0.009) but in second group there was not statistically significant differences between heart rate (*P*=0.345).

***Conclusion:*** Although Ventolin can cause changes in QTc, Ventolin with 0.15 mg/kg/dose in comparison with 0.1 mg/kg/dose does not cause significant changes in QTc.

## Introduction

Patients with respiratory disease frequently use high doses of β2-agonists. β2-agonists relax vascular smooth muscle and cause peripheral vasodilation, with reflex cardiac stimulation leading to tachycardia; direct stimulation of cardiac β1- and β2-adrenoreceptors also contributes to an increase in heart rate and contractility.^1^ β2-agonists can cause T wave changes and prolonged QTc interval on the electrocardiogram.^2^ The World Health Organization (WHO) recommended name for albuterol base is salbutamol. For children, initial dosing should be based upon body weight (0.1 to 0.15 mg/kg per dose). Dosing should not be more than 2.5 mg three to four times daily by nebulization.^3^ A prolonged QT interval is a marker for the potential of ventricular tachyarrhythmia like torsades de pointes and a risk factor for sudden death. The QT interval is commonly measured in lead II for evaluation of serial electrocardiograms, with leads I and V5 being comparable alternatives to lead II.^4^ The main focus of this investigation was on the safety of inhaled β2-agonists in the treatment of respiratory disease in children. In this study, we used Ventolin as a β2-agonist and investigated its effect on QTc and heart rate. Also comparison between two different doses of Ventolin (0.15 mg/kg/dose versus 0.1 mg/kg/dose) was assessed.

## Materials and Methods

The present investigation is a descriptive cross-sectional prospective study from a leading cardiac center in southwest of Iran (Golestan hospital, Ahvaz Jundishapur University Of Medical Sciences) during March 2013 to February 2014. A total of 192 patients aged between 2 months and 15 years with respiratory disease referred to pediatric emergency ward which was candidate for treatment with Ventolin by physicians were divided into two groups. Since dosage of inhaled Ventolin ranges between 0.1-0.15 mg/kg/dose we divided the patients randomly into two groups. The first group consisted of 128 patients who received 0.15 mg/kg/dose nebulized Ventolin and second group consisted of 64 patients received 0.1 mg/kg/dose Ventolin in three times each 20-minute interval. Including criteria consist of children aged between 2 months and 15 years, pulse oximetry above 92% in admission, not prior use of β2-agonists and corticosteroid drugs, no chronic disease such as renal, cerebral and metabolic, not use any medication that could affect the electrocardiogram. Patients with electrolyte disturbances were excluded from this study. Normal ranges for potassium and sodium were considered as 3.5-5 meq/L, 130-150 meq/L and 8-10 mg/dl for calcium. Heart rate before (HR1) and after (HR2) nebulized Ventolin and also QTc was assessed from electrocardiogram. Beginning of the Q to the end of the T wave in electrocardiogram is the QT interval. Because of patients’ heart rate greater than 75 bps Fridericia formula was used instead of Bazett’s formula. QT interval corrections use Fridericia formula, defined as the observed QT interval divided by has cube-root of R-R interval in second if the standard clinical correction is to used Bazett’s formula, defined as the QT interval divided by the square root of the R-R interval in seconds.^5^ Electrocardiographic recording was performed at all leads but QTc interval was measured in lead II for evaluation of serial electrocardiograms. Heart rate was taken from cardiac monitoring and electrocardiogram. Data were analyzed by SPSS software version 17. Differences between two groups were assessed using a paired student’s *t* test.

## Results

Of 192 patients who had inclusion criteria, 142 cases (74%) were male and 50 cases (26%) were female. Mean age of patients was 4.93 ± 3.87 years. Demographic data were shown in Table 1. In 170 patients (88.5%), QTc2 were higher than QTc1 and in 19 patients (9.9%) QTc2 were equal to QTc1 and 3 patients (1.6%) showed lower QTc2 than QTc1. Three patients out of 192 showed an unexpected QTc after receiving nebulized Ventolin that all were in first group. One of them had QTc greater than 450 milliseconds and two of them had QTc less than 450 milliseconds but differences between QTc2 and QTc1 were greater than 50 milliseconds all those three patients did not show any arrhythmia or any other symptoms. In first group, mean QTc1 was 352 ± 32 milliseconds and mean QTc2 was 376 ± 33 milliseconds. In second group, mean QTc1 was 358 ± 23 milliseconds and mean QTc2 was 372 ± 24 milliseconds. There was statistically significant differences between QTc1 and QTc2 in each groups (*P *< 0.005). According to independent T test, mean QTc1 interval in both groups were almost equal and there was not any significant differences (*P* = 0.2). The same as QTc1, QTc2 in both groups did not show any significant differences (*P* = 0.47). In first group there was statistically significant differences between HR1 and HR2 (*P* = 0.009) but in second group there was no statistically significant differences between HR1 and HR2 (*P* = 0.345). To compare both groups for mean HR1 there was not any statistically significant differences (*P* = 0.1) but after nebulized Ventolin mean HR2 in first group were significantly greater than the second group (*P* = 0.02). Changes in mean QTc and mean heart rate, before and after receiving Ventolin in two groups were shown in Table 2.

**Table 1:** Demographic data of patients in two groups

**Groups**	**Sex**	**Mean age (year)**	**Mean weight (kg)**
Group 1^a^	Male: 95 (74.2%) Female: 33 (25.8%)	4.07 ± 3.25	17.33 ± 8.34
Group 2^b^	Male: 47 (73.4%) Female: 17 (26.6%)	6.67 ± 4.42	26.60 ± 16.90

^a^Patients who received 0.15 mg/kg/dose nebulized Ventolin in three times each 20-minute interval. ^b^patients who received 0.1 mg/kg/dose nebulized Ventolin in three times each 20-minute interval.

**Table 2:** Changes in mean QTc and mean heart rate before and after receiving Ventolin

**Groups**	**QTc1 (ms)**	**QTc2 (ms)**	**QTc2-QTc1 (ms)**	**HR1 (beats/min)**	**HR2 (beats/min)**	**HR2-HR1 (beat/min)**
Group 1	352±32	376±33	23±15^a^	139±22	142±21	2.9±12.5^a^
Group 2	358±23	372±24	14±10^a^	133±24	134±23	1.2±10.2^b^

^a^P < 0.05; ^b^ *P* = 0.345

## Discussion

This study reflects the effect of Ventolin on electrocardiogram that showed Ventolin can prolong QTc interval and increase heart rate. Use of β2-agonists in children is common, either for acute management or for prophylaxis of exercise induced bronchospasm. Salbutamol inhalation decreased parasympathetic and increased sympathetic cardiovascular autonomic balance.^6^ Inhaled β2-agonists can increase heart rate and systolic blood pressure, decrease diastolic blood pressure and prolong QTc interval in healthy volunteers and patients with asthma and chronic obstructive pulmonary disease.^7^ β2-agonist inhalation at doses recommended for bronchodilating therapy have shown that salbutamol and fenoterol produce an increase in heart rate and a decrease in diastolic blood pressure.^8^ These confirm the result of our study in increasing heart rate of patients received Ventolin especially with higher doses. Like other systemic effects, changes in heart rate, QTc interval and T wave occurs after the use of long-term inhaled β2-agonist.^9^ A study showed that the increase of the QTc is more significant with the use of a standard dose of albuterol (0.15 mg/kg) in comparison with low dose albuterol (0.075 mg/kg) plus ipratropium.^10^ Although there is controversy in the effect of hypoxia on QTc,^11^ in this study we enrolled patients without hypoxia. Furthermore, because of the effect of electrolyte imbalances on electrocardiogram,^12^ patients with normal electrolyte ranges were enrolled. In addition, patients with a history of consumption of corticosteroids and other drugs that could affect electrocardiogram were excluded.^13^ Also, In contrast with Rosenkranz et al that found minimal changes in QTc intervals after using a high dose of β2-agonist, results of this investigation showed significant changes in QTc interval after using nebulized Ventolin.^14^ Since certain conclusions necessitate researches that evaluate long term effect of β2-agonist on cardiac autonomic function and observe whether changes are permanent or temporary, we recommend furthermore widespread investigations in this field.

## Conclusion

According to the result of this investigation nebulized Ventolin can increase QTc interval. Ventolin with 0.15 mg/kg/dose in comparison with 0.1 mg/kg/dose did not make significant changes in QTc. A higher dose of Ventolin could predispose children to tachycardia so arrhythmias following Ventolin intake should be considered and cardiac monitoring in children receiving Ventolin is recommend.

## Ethical approval

This study has been approved by Ahvaz Jundishapur Medical Sciences ethical committee (Thesis number: U-92138).

## Competing interests

Authors declare no conflict of interests in this study.

## Acknowledgments

We acknowledge all the parents and patients for their commitment to the study. The authors would like to thank the Deputy of Research and Technology of Ahvaz Jundishapur University of Medical Sciences for supporting this research.
